# Supplementary figures and images for: Conserved structural elements specialize ATAD1 as a membrane protein extraction machine
Source: eLife. 2022 May 12;11:e73941. doi: 10.7554/eLife.73941 (PMC9273213; doi:10.7554/eLife.73941)

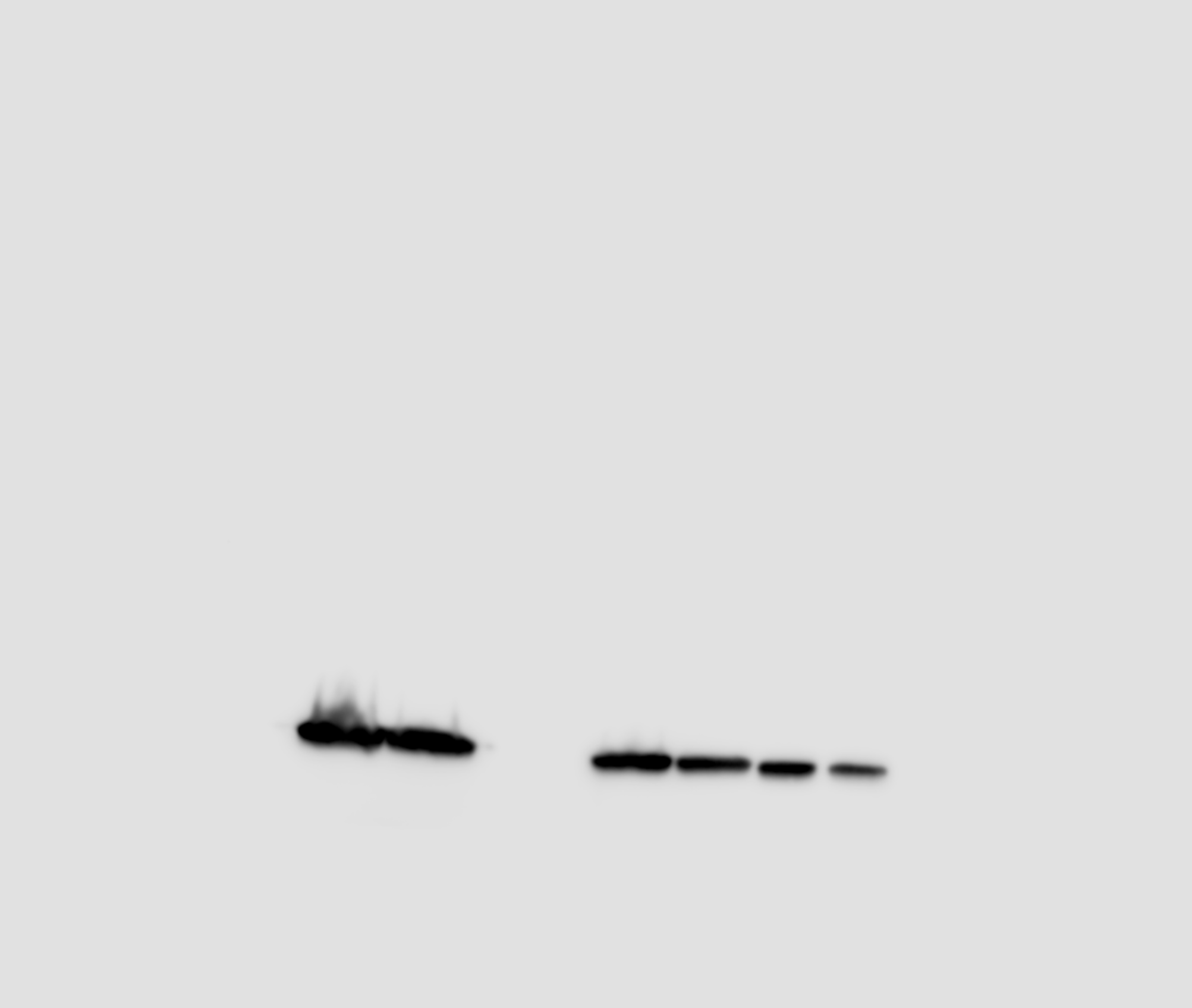

Supplement: Figure 3—source data 1. [file elife-73941-fig3-data1.zip › Source_data/Figure3_figure_supplement1_original_blot_low_exposure.tiff]

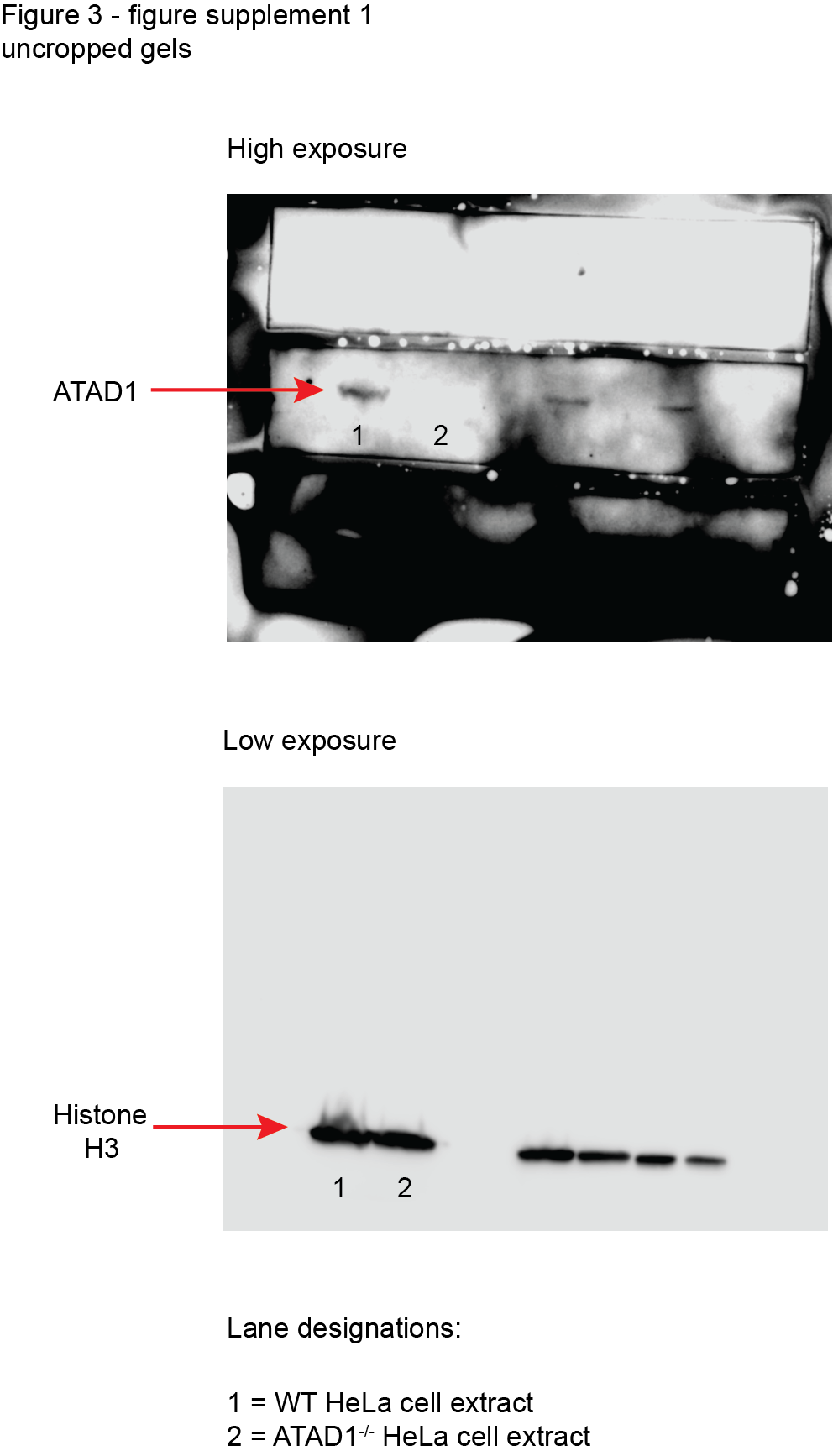

Supplement: Figure 3—source data 1. [file elife-73941-fig3-data1.zip › Source_data/Figure3_figure_supplement1_uncropped_gel.png]

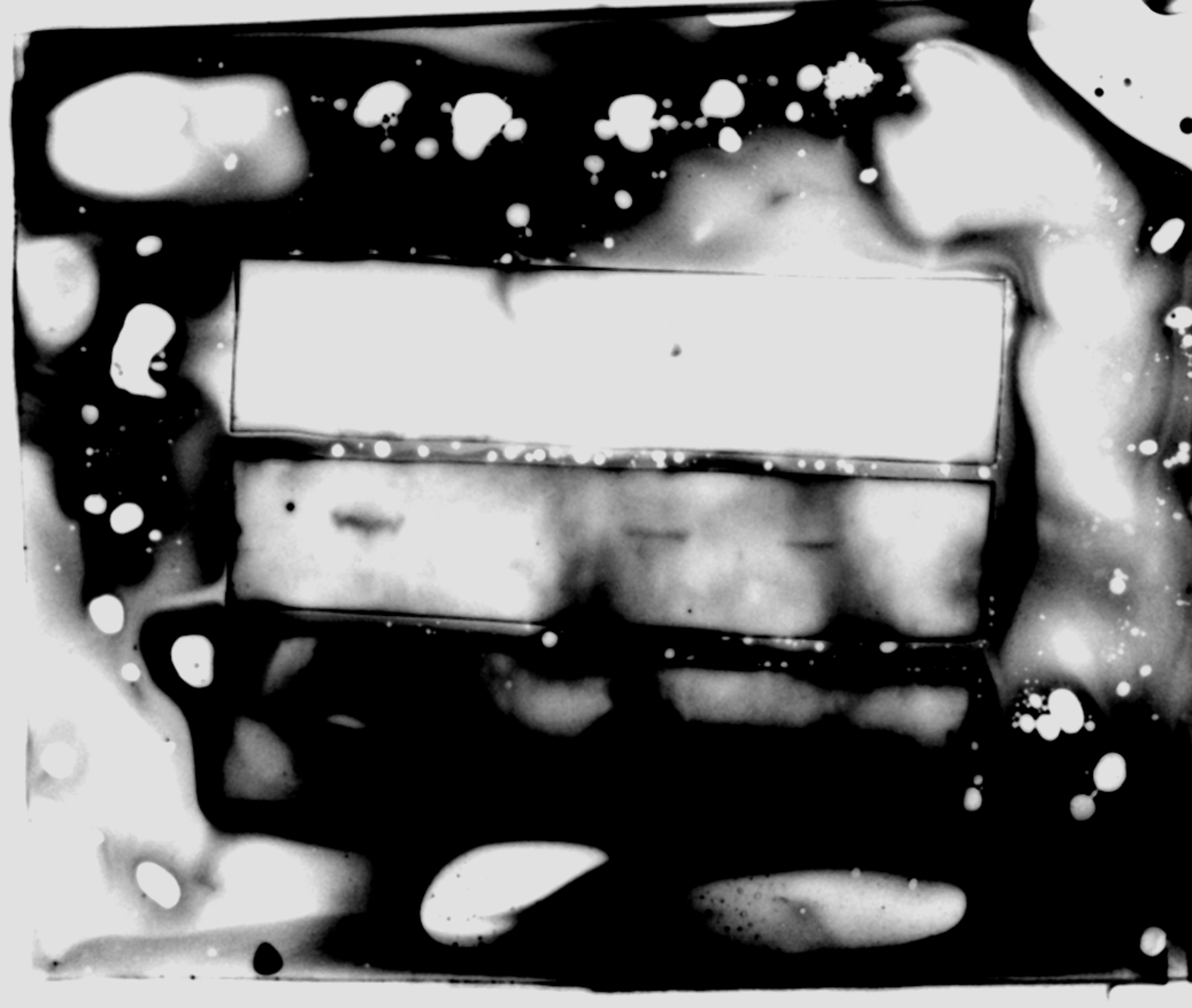

Supplement: Figure 3—source data 1. [file elife-73941-fig3-data1.zip › Source_data/Figure3_figure_supplement1_original_blot_high_exposure.tiff]
